# Controlling the Thermoelectric Behavior of La-Doped SrTiO_3_ through Processing and Addition of Graphene Oxide

**DOI:** 10.1021/acsami.2c14408

**Published:** 2022-11-22

**Authors:** Dursun Ekren, Jianyun Cao, Feridoon Azough, Demie Kepaptsoglou, Quentin Ramasse, Ian A. Kinloch, Robert Freer

**Affiliations:** † Department of Materials, University of Manchester, Oxford Road, ManchesterM13 9PL, U.K.; ‡ Department of Metallurgical and Materials Engineering, Iskenderun Technical University, İskenderun31200, Hatay, Turkey; § Key Laboratory of LCR Materials and Devices of Yunnan Province, School of Materials Science and Energy, Yunnan University, Kunming650500, China; ∥ SuperSTEM Laboratory, SciTech Daresbury Campus, Daresbury, WarringtonWA4 4AD, U.K.; ⊥ Department of Physics, University of York, YorkYO10 5DD, U.K.; # School of Chemical and Process Engineering, University of Leeds, LeedsLS2 9JT, U.K.; ∇ Henry Royce Institute and National Graphene Institute, University of Manchester, Oxford Road, ManchesterM13 9PL, U.K.

**Keywords:** SrTiO_3_, thermoelectric, grain boundary, graphene oxide, composite

## Abstract

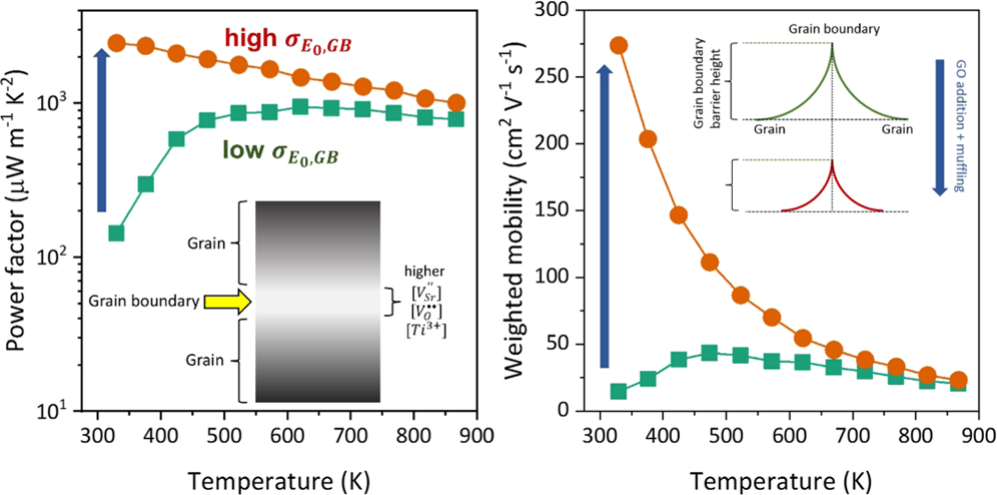

The addition of graphene has been reported as a potential route to enhance the thermoelectric performance of SrTiO_3_. However, the interplay between processing parameters and graphene addition complicates understanding this enhancement. Herein, we examine the effects of processing parameters and graphene addition on the thermoelectric performance of La-doped SrTiO_3_ (LSTO). Briefly, two types of graphene oxide (GO) at different oxidation degrees were used, while the LSTO pellets were densified under two conditions with different reducing strengths (with/without using oxygen-scavenging carbon powder bed muffling). Raman imaging of the LSTO green body and sintered pellets suggests that the added GO sacrificially reacts with the lattice oxygen, which creates more oxygen vacancies and improves electrical conductivity regardless of the processing conditions. The addition of mildly oxidized electrochemical GO (EGO) yields better performance than the conventional heavily oxidized chemical GO (CGO). Moreover, we found that muffling the green body with an oxygen-scavenging carbon powder bed during sintering is vital to achieving a single-crystal-like temperature dependence of electrical conductivity, implying that a highly reducing environment is critical for eliminating the grain boundary barriers. Combining 1.0 wt % EGO addition with a highly reducing environment leads to the highest electrical conductivity of 2395 S cm^–1^ and power factor of 2525μW m^–1^ K^–2^ at 300 K, with an improved average *zT* value across the operating temperature range of 300–867 K. STEM-EELS maps of the optimized sample show a pronounced depletion of Sr and evident deficiency of O and La at the grain boundary region. Theoretical modeling using a two-phase model implies that the addition of GO can effectively improve carrier mobility in the grain boundary phase. This work provides guidance for the development of high-performance thermoelectric ceramic oxides.

## 1 Introduction

Direct conversion of waste heat to electricity makes thermoelectric (TE) power generators an attractive technology to reduce the use of fossil fuels. TE performance is typically characterized by the dimensionless figure of merit, *zT* = (σ*S*^2^*/*κ)*T*, where σ, *S*, and κ are the electrical conductivity, Seebeck coefficient, and thermal conductivity, respectively. The dependence of these parameters on carrier concentration complicates routes to maximizing TE performance; nonetheless, high σ and *S*, in addition to low κ, are necessary to achieve the maximum *zT* (*zT*_max_) values.^1^ One approach to realizing high thermoelectric performance is based on the work of Dresselhaus et al.,^2^ which indicated that nanostructuring could lead to significant improvements in *zT*.

Among the materials explored for use in TE generators, oxides have emerged as promising candidates for high-temperature TE applications due to their good thermal and chemical stability, together with the abundance and low cost of raw materials.^3^ SrTiO_3_ (STO), as an n-type TE material, has been of particular interest as the power factor (σ*S*^2^) of the undoped oxide is comparable to that of commercial TE materials, e.g., Bi_2_Te_3_.^4^ However, the high thermal conductivity of STO, κ > 3 W m^–1^ K^–1^ at 1000 K, limits its overall TE performance. The general nanostructuring approach for reducing thermal conductivity is not as effective for STO as other TE materials because of the small mean free path for phonons in this material (≈2 nm at 1000 K).^5^ It has been demonstrated that grains smaller than 55 nm are required to achieve a significant reduction of κ.^5^ Another effective, and more recent approach for reducing the thermal conductivity of STO is to develop nanocomposites by embedding nanostructured secondary phases (fillers) into the STO matrix to increase the scattering of phonons by interfaces.^6−9^

Single-atom-thick graphene flakes have been identified as promising additives for developing TE composites because of their high electrical conductivity^10^ and reasonably high Seebeck coefficient (a few μV K^–1^ for single-layered graphene to ∼100 μV/K^–1^ for multilayered graphene).^11,12^ Recently, the observation by Lin et al.,^7^ that addition of graphene extended the thermoelectric temperature range of operation (or TE window) and improved the TE properties of SrTiO_3_/graphene composites, heightened interest in functional graphene composites for thermoelectric applications. Zebarjadi et al.^13^ demonstrated, theoretically, that inclusion of a metallic secondary phase could enhance TE properties, and indeed, such an effect could be achieved by the addition of graphene-based materials to a ceramic matrix. Subsequently, this behavior was observed for STO materials by the addition of graphene^7^ and metallic particles (Fe or Cu)^14^ to the STO matrix. The only potential disadvantage of incorporating graphene in TE composites is the extremely high thermal conductivity of graphene (∼5000 W m^–1^ K^–1^^15^); however, the presence of a two-dimensional material at grain boundaries provides a way to enhance phonon scattering and thereby reduce the overall thermal conductivity.^16^

In recent years, there have been numerous investigations into the effects of graphene-based fillers on the TE properties of functional composites made of inorganic materials: Bi_2_Te_3_,^17,18^ CoSb_3_,^19^ ZnO,^20^ (Bi,Sb)_2_Te_3_,^21^ Cu_2_Se,^22^ Yb*_y_*Co_4_Sb_12_ and Ce*_y_*Fe_3_CoSb_12_,^23^ Cu_2–*x*_S,^24^ TiO_2_,^25^ La-doped SrTiO_3_,^7^ pure SrTiO_3_,^8^ Nb-doped SrTiO_3_,^9,26^ Nb-doped A-site-deficient SrTiO_3_,^27^ and La- and Nb-doped A-site deficient SrTiO_3_.^28^ One common observation has been the reduction of thermal conductivity, regardless of the matrix material, while enhancing electrical conductivity.^29^ This led to improved *zT* values in all cases;^7−9,17−25,27,28^ for example, ∼130 and ∼120% improvements in *zT* were reported for CoSb_3_^19^ and Cu_2_Se,^22^ respectively. We have previously demonstrated that La-doped SrTiO_3_ (LSTO) composites containing graphene, which were densified via conventional pressureless sintering in an Ar–5%H_2_ atmosphere, exhibited significantly improved maximum *zT*_max_ in addition to temperature-stable *zT* values, in comparison to the base composition;^7^ the *zT*_max_ was improved from 0.26 to 0.36 at 1023 K and it was ≥0.26 from room temperature to 1023 K. In a later study, Feng et al.^8^ showed that the addition of reduced graphene oxide (RGO) promoted the formation of oxygen vacancies at grain boundaries (GBs), which led to increased carrier concentrations and higher σ*S*^2^ values for pure SrTiO_3_. Similarly, Okhay et al.^9^ showed that the *zT* value of Nb-doped SrTiO_3_ could also be improved by the addition of RGO, leading to a maximum *zT* value of 0.29 at 1160 K.

In the case of single crystalline and polycrystalline La-doped SrTiO_3_, Moos and Härdtl^30^ showed that both exhibited similar temperature dependencies of electrical conductivity above 400 K, following T^–1.6^ dependence. However, the effect of grain boundaries on conduction was clear; there was a significant difference in the carrier mobility, and thus, the electrical conductivity of the ceramics was much lower than that of the single crystalline sample. These results, which were supported by later work on SrTiO_3_,^31^ demonstrated that the presence of GBs was detrimental to the electronic transport properties of the material and consequently the overall performance. More recently, it was suggested that the improved TE properties in polycrystalline SrTiO_3_/graphene composites and the occurrence of single-crystal-like conduction behavior was due to the decrease in the height of double Schottky barriers at GBs by the formation of oxygen vacancies in the vicinity of GBs.^32,33^ This mechanism overcomes the negative effect of grain boundaries on the conduction of electrons (which typically lowers the charge carrier mobility) and enables single-crystal-type behavior in polycrystalline ceramics. However, many LSTO ceramics/composites were processed under a variety of conditions (e.g., oxygen partial pressure, temperature, etc.), which affected the properties of the resulting materials, thereby complicating the unambiguous interpretation of the effects of graphene on the properties of the ceramic. For instance, metallic/single-crystal-like electrical conduction is not observed in all LSTO/graphene composites;^7−9,27,28^ only those in which either an oxygen-scavenging bed or high vacuum (10^–3^ torr) were used to create a low oxygen partial pressure environment.^7,9,33,34^ In our recent work,^34^ we prepared Sr_0.9_La_0.08_TiO_3_ under a series of increasingly reducing atmospheres and showed that metallic/single-crystal-like behavior was only achieved by the use of the most reducing atmosphere that provided the necessary control of charge transport at grain boundaries.

In view of the uncertainty in the literature about the impact of different processing environments, we systematically investigated the effects of key parameters, including the type of graphene addition, on the thermoelectric performance of Sr_0.9_La_0.08_TiO_3_. Herein, we first demonstrate that sintering in a simple reducing environment (Ar/5%H_2_ atmosphere) with addition of electrochemically produced graphene oxide (EGO) is beneficial for improving the electrical conductivity but has limited impact on the temperature dependency of electrical conductivity. In contrast, the use of additional muffling (surrounding the samples with 5 wt % GNP added LSTO) powder leads to enhanced electrical conductivity and single-crystal type behavior; the effect is more pronounced with addition of EGO. It was also shown that the ratio of weighted mobility to lattice thermal conductivity (μ_w_/κ_lattice_) can be increased tenfold solely by the muffling approach when processing LSTO–GO composites. This work decouples and unambiguously identifies the effects of different processing environments (specifically the use of muffling) and the addition of graphene oxide on the TE properties of LSTO ceramics; it can be used to guide the development of other high-performance oxide TE materials.

## 2 Materials and Methods

### 2.1 Preparation of La-Doped SrTiO_3_/Graphene Oxide Hybrid Powders

A-site deficient La-doped SrTiO_3_ powders (Sr_0.9_La_0.08_TiO_3_ = LSTO) were prepared using the same experimental approach we previously used;^34^ after final milling, the average particle size was ∼590 nm (Figure S1a, supporting material). For the composites, two types of graphene oxide were used: chemical graphene oxide (CGO) prepared using Hummers’ method^35,36^ and electrochemical graphene oxide (EGO, Figure S1b, supporting material) prepared using a two-step electrochemical intercalation and oxidation approach.^37^ The as-prepared CGO and EGO were subject to probe sonication (Fisherbrand Q700 Sonicator) for 1 h in an ice water bath to reduce the flake size to less than 1 μm.

An electrostatic assembling method was used to mix the GO nanosheets with the LSTO powder. Briefly, 5 g of LSTO powder was dispersed in 500 mL of water using bath sonication for 1 h. To positively charge the surface of LSTO nanoparticles, the pH value of the LSTO dispersion was adjusted to 3 using acetic acid before the sonication procedure. Then, the GO dispersion was added dropwise to the as-formed LSTO dispersion while stirring, enabling the negatively charged GO nanosheets to attach to the positively charged LSTO surfaces via electrostatic interaction. The mixed dispersion was stirred for 30 min; the LSTO/GO hybrids were then collected using vacuum filtration and dried at 80 °C for 12 h. For the samples to be densified without using a sacrificial carbon bed muffle, the EGO in the hybrid powders was reduced using hydrazine vapor at 100 °C for 3 h before sintering; the samples prepared with this reduced EGO are labeled REGO.

### 2.2 Pressing and Sintering of La-Doped SrTiO_3_/Graphene Oxide Composites

The hybrid powders were uniaxially pressed to form discs of 15 or 20 mm diameter and 5 mm height and sintered at 1700 K using two different environments. The LSTO/GO hybrids sintered directly under an Ar/5% H_2_ atmosphere with 150 mL/min flow rate (in the presence of a powder muffling) are labeled with HA, while the samples sintered under Ar/5% H_2_ flow in the presence of an LSTO + 5 wt % graphene nanoplatelet (GNP) powder covering are labeled with M-HA. The oxygen-scavenging powders were prepared by vibratory milling of LSTO powder with 5 wt % graphene nanoplatelets for 5 h. About 15 g of LSTO + 5 wt % GNP powders were used for each sintering experiment in the presence of oxygen-scavenging powders. The optimized sintering times were 12 h for HA and 24 h for M-HA. The as-sintered pellets were cut into bar- or disc-shaped samples for further measurements. Table 1 summarizes the composition, processing condition, and reference code for each sample.

**Table 1:** Compositions, Processing Conditions, and Codes for Sr_0.9_La_0.08_TiO_3_ (LSTO)–Graphene Oxide Samples

composition	processing condition	sample code	relative density (%)
LSTO	in H_2_–Ar	LSTO-HA	94.0
LSTO	in H_2_–Ar and muffled in sacrificial powder	LSTO-M-HA	89.3
LSTO + 0.5 wt % REGO	in H_2_–Ar	0.5REGO-HA	98.0
LSTO + 1.0 wt % REGO	in H_2_–Ar	1.0REGO-HA	96.4
LSTO + 1.5 wt % REGO	in H_2_–Ar	1.5REGO-HA	95.1
LSTO + 1.0 wt % EGO	in H_2_–Ar	1.0EGO-HA	97.1
LSTO + 1.0 wt % EGO	in H_2_–Ar and muffled in sacrificial powder	1.0EGO-M-HA	96.4
LSTO + 1.0 wt % CGO	in H_2_–Ar and muffled in sacrificial powder	1.0CGO-M-HA	91.3

### 2.3 Characterization

X-ray diffraction (XRD) patterns for the samples were collected using a Philips X’Pert diffractometer with a Cu Kα source (λ_Cu Kα_ = 1.540598 Å). A continuous scan between 20 and 100° was recorded using 0.0167° step size and a dwell time of 6 s per step. XRD patterns were analyzed with the aid of X’Pert High Score software for phase determination. For microstructural analysis, a TESCAN MIRA3 SC field emission gun–scanning electron microscopy (FEG–SEM) fitted with an energy dispersive spectroscopy (EDS) detector was employed. Raman spectroscopy was conducted using a Renishaw InVia Raman spectrometer, with an excitation laser wavelength of 633 nm and a spot size of 2 μm.

Samples for transmission electron microscopy (TEM) and scanning TEM (STEM) observation were prepared by standard crushing procedures; sintered disks were crushed to a powder using an agate mortar and pestle. Grains of individual powders were dispersed in chloroform, dropped onto a copper grid covered with a holey carbon film, and then dried in air. The structures of the grains and grain boundaries were investigated by selected area electron diffraction (SAED) and high resolution TEM (HRTEM) techniques using an FEI FEGTEM (Tecnai G2, Hillsboro, OR) operating at 300 kV. More detailed high-angle annular dark-field (HAADF) STEM imaging and EELS characterization were performed using a Nion UltraSTEM 100 aberration-corrected dedicated scanning transmission electron microscope equipped with a Gatan Enfina spectrometer, operating at 100 kV. The convergence semiangle and the HAADF inner outer collection angles were 31 mrad and 86–200 mrad, respectively. The EELS collection angle was 36 mrad. The EELS data were denoised by the Principal Component Analysis HREM-Research MSA plugin for Digital Micrograph.^38^

A ULVAC ZEM-3 system was utilized for the simultaneous measurement of electrical conductivity (σ) and the Seebeck coefficient (*S*) from 300 to 900 K in a low-pressure helium atmosphere. The thermal conductivity (κ) of the samples was obtained indirectly from κ = α × *d* × *C*_p_, where α is the thermal diffusivity, *d* is the density, and *C*_p_ is the specific heat capacity. A Netzsch LFA-457 Laser Flash apparatus was used to determine the thermal diffusivity, and the density was determined from mass and dimension measurements. The heat capacity was measured in an Ar atmosphere using a Netzsch DSC 404 F1 Pegasus facility.

## 3 Results and Discussion

Our recent work on LSTO ceramics showed that the processing approach has significant effects on the electronic transport behavior of the ceramics.^34^ It was clear that the presence of sacrificial powder was beneficial for reducing the height of potential barriers at GBs and thus leading to significant improvement in electric conductivity without degrading the Seebeck coefficient. To further improve the thermoelectric properties of the LSTO-based materials, composites were prepared with GO obtained from different approaches and the bulks were produced under two different sintering environments (HA and M-HA). Based on the earlier results,^34^ the samples showing thermally activated conduction behavior are inferred to have higher potential barriers and hence more resistive grain boundaries (RGBs) with respect to the samples that exhibit metallic-type electrical conductivity behavior, and therefore lower potential barriers, which are again inferred to result from more conductive grain boundaries (CGBs).

**Figure 1:**
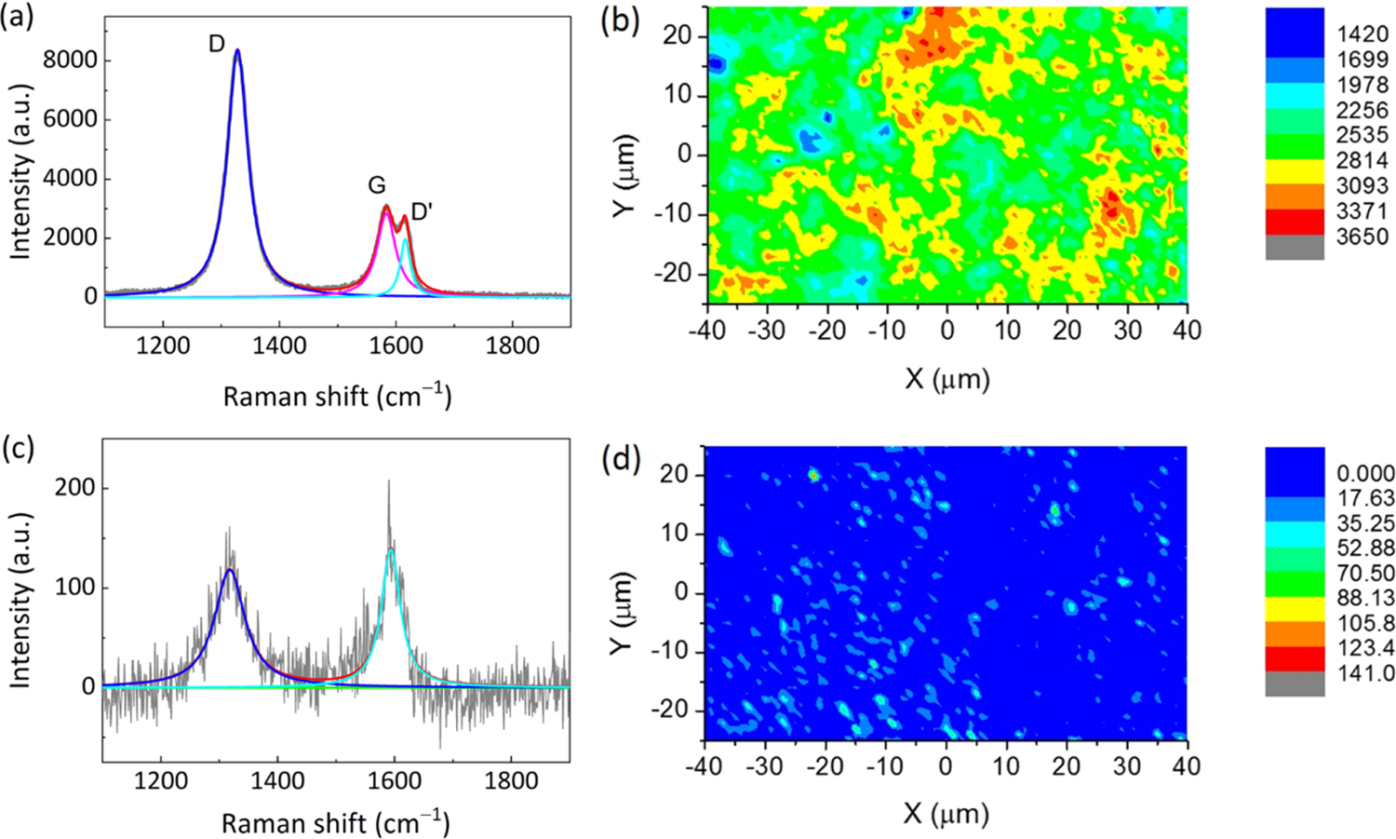
Raman spectroscopy characterization of REGO in LSTO matrix before and after sintering: (a) typical Raman spectrum and (b) G band intensity mapping of the hybrid powder of 1 wt % REGO and LSTO before sintering; (c) a typical Raman spectrum from where carbon was present and (d) G band intensity mapping of the sintered sample prepared from this hybrid powder.

On the basis of a range of trial experiments, it was clear that addition of EGO/REGO yielded higher thermoelectric performance than addition of chemically prepared graphene oxide (CGO); supporting evidence will be presented later. We therefore focus on the effects of addition of EGO/REGO and initially on the properties of LSTO ceramics with RGBs. Different loadings of REGO (0.5, 1.0, and 1.5 wt %) and EGO (1.0 wt %) were introduced into the LSTO ceramic matrix and samples sintered under H_2_–Ar flow alone. Raman spectroscopy was used to investigate the change of REGO before and after sintering. Figure 1a shows a typical Raman spectrum collected from the hybrid powder of LSTO with 1.0 wt % REGO before sintering. The spectrum shows intense D, G, and D’ bands, with a D-to-G band intensity ratio (*I*_D_/*I*_G_) of 2.96, suggesting sufficient reduction and restoration of the graphene lattice structure after hydrazine vapor reduction. The G band intensity mapping (Figure 1b) of the pressed pellet before sintering indicates the abundance of REGO in the entire mapped area. In contrast, the densified (sintered) pellet shows a much lower abundance of REGO as the G band intensity is absent across the majority of the mapped area (Figure 1d). A typical Raman spectrum collected from the REGO-rich area (Figure 1c) shows broad D and G bands with a *I*_D_/*I*_G_ ratio of 0.86, suggesting a more defective structure compared with the REGO before densification (*I*_D_/*I*_G_ = 2.96). Generally, high-temperature annealing in inert or reducing atmospheres will further reduce the material and restore the honeycomb lattice structure of REGO. However, the Raman mapping results clearly indicate degradation/consumption of REGO during the high-temperature sintering process.

It is well established that heat treatment of STO at temperatures exceeding 1273 K leads to the formation of doubly ionized oxygen vacancies, each with two electrons, as shown in eq 1.^8^ The as-formed oxygen is further consumed by the reducing atmosphere (e.g., H_2_–Ar atmosphere), generating more oxygen vacancies, and leading to a higher carrier concentration. Hence, the added graphene flakes inside the LSTO ceramic compacts are able to act as oxygen scavengers, which react with the as-formed oxygen (eq 2). In other words, the graphene flakes in the LSTO ceramic compacts are consumed during the sintering process, as confirmed by the Raman mapping results (Figure 1) and SEM micrographs from fracture surfaces (Figure S2, supporting material). This sacrificial reaction of the graphene oxide with lattice oxygen promotes the formation of oxygen vacancies, as evidenced by the increase of LSTO lattice parameter at higher graphene loadings (Figure S3, supporting material, shows changes in 2θ in XRD patterns). Possible reactions that occur during the sintering process are given below:

(i) The formation of an oxygen vacancy and two electron carriers via loss of oxygen from the lattice
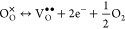
1
(ii) The consumption of the as-formed oxygen via reaction with graphene oxide
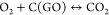
2
(iii) Released oxygen reacts with the hydrogen from the sintering atmosphere, particularly for the case when graphene is not added
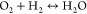
3

Both reactions 2 and 3 promote the formation of more oxygen vacancies and thus an increased level of reduction of the LSTO ceramic.

**Figure 2:**
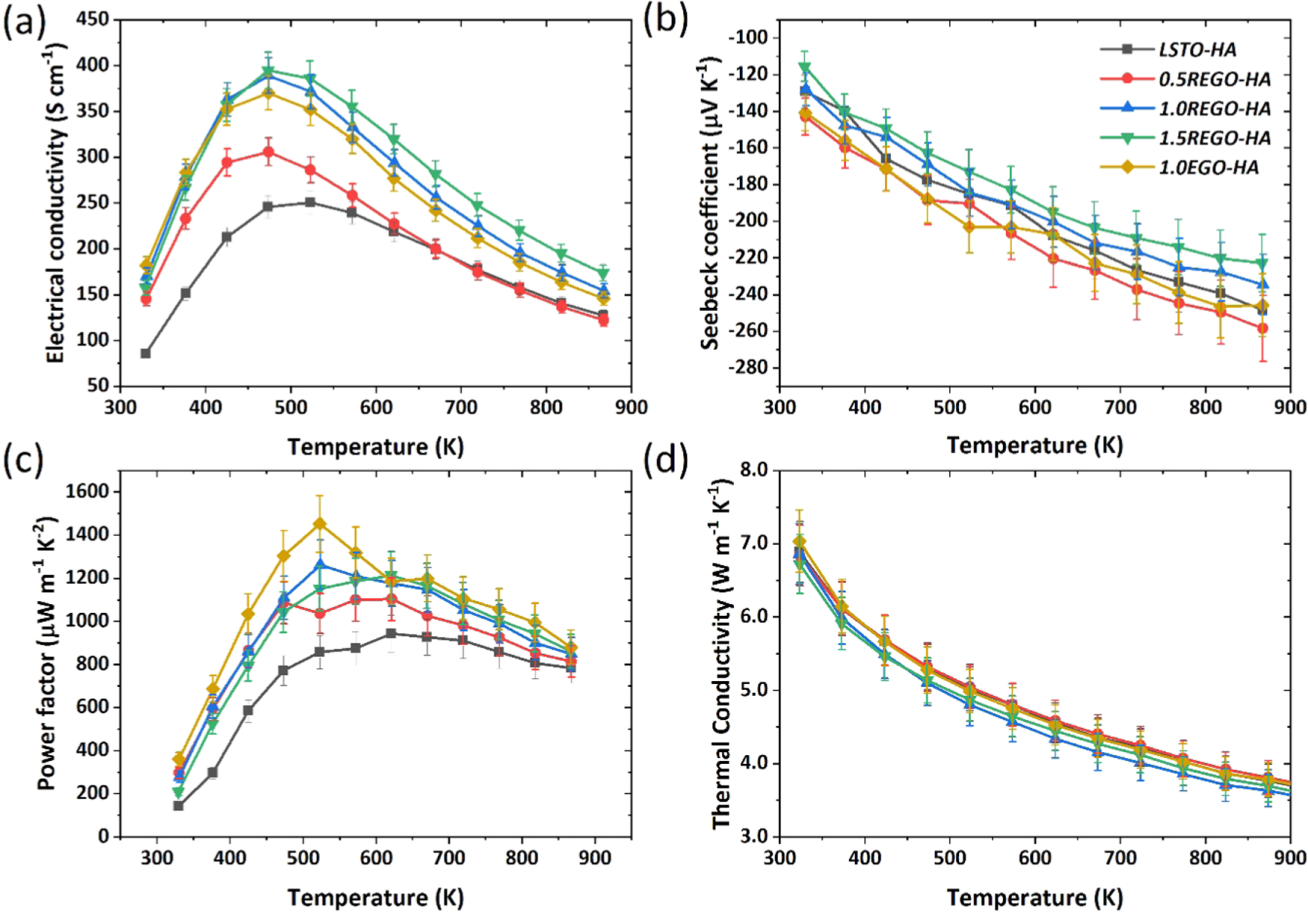
Temperature dependency of the thermoelectric properties of LSTO-based composites with resistive grain boundaries: (a) electrical conductivity, (b) Seebeck coefficient, (c) power factor, and (d) thermal conductivity. The samples were sintered under H_2_–Ar flow in the absence of sacrificial powder.

All of the graphene-containing samples were more than 90% dense, and most had more than 95% theoretical density (Table 1 and Figure S4, supporting material). XRD analysis confirmed the cubic perovskite structure with *Pm*3̅*m* space group symmetry (Figures S3 and S5, supporting material). After sintering without muffling, the thermoelectric properties of LSTO ceramics prepared from hybrid powders with different levels of REGO loading were determined (Figure 2). All samples exhibit thermally activated electron transport behavior with maximum electrical conductivity occurring at around 475 K (Figure 2a), suggesting that the grain boundaries remain resistive (high potential barriers) at low temperatures. Nevertheless, with the addition of 1.0 wt % REGO, the electrical conductivity at 523 K increases noticeably from 250.5 to 371.7 S cm^–1^. At the higher REGO loading of 1.5 wt %, the σ increases slightly, reaching 386.1 S cm^–1^ at 523 K. Even though the grain boundaries remain resistive at low temperatures, the results clearly indicate that the addition of GO enhances σ in the LSTO ceramics. This supports earlier findings where a similar temperature dependency of conductivity was reported.^8,9,27^ Combining this with our XRD and Raman spectroscopy results suggests that the enhancement of σ can be attributed to the formation of additional oxygen vacancies through the sacrificial reaction between added REGO and lattice oxygen (eq 2).

The LSTO ceramics prepared with and without GO addition all show negative Seebeck coefficient values, confirming the n-type nature of the samples (Figure 2b). The effect of GO addition on *S* is much less evident than its impact on σ. All of the samples exhibit comparable |*S*| values, except for a slight reduction in |*S*| for the sample with the highest REGO loading of 1.5 wt %. As a result of the combined effects of the large increase in σ and the minor changes in *S*, there is still a net improvement in σ*S*^2^ upon the addition of GO (Figure 2c). At 523 K, σ*S*^2^ increases from 857.9 μW m^–1^ K^–2^ (sample without GO) to 1452.7 μW m^–1^ K^–2^ for samples with 1.0 wt % EGO addition. It can be seen from Figure 2c that, within the uncertainty in the measurements, the samples with the same levels of EGO and REGO additions exhibit similar maximum power factor values when sintering is performed in the absence of sacrificial powder. In contrast, they are much higher for EGO-added samples sintered in the presence of sacrificial powder as a result of enhanced carrier transport. The effect of the thermally activated conduction mechanism is also illustrated by the temperature dependency of power factor values, which initially rise with temperature and then decrease above 523 K due to the reduction of the conductivity at high temperatures (Figure 2c).

The thermal conductivity for samples with RGBs (Figure 2d) also exhibited a T^–1^ type temperature dependency, which is typical for crystalline materials^39^ as Umklapp phonon-phonon scattering becomes the dominant scattering mechanism above the Debye temperature, which was reported as 513 K for SrTiO_3_.^40^ The κ of the base LSTO reference sample decreased from 6.89 to 3.54 W m^–1^ K^–1^ between 323 and 973 K. The addition of REGO led to a small reduction in κ; the lowest κ values were obtained with 1.0 wt % REGO addition, but differences are close to the level of uncertainty in the data. This reduction in κ with the addition of REGO is modest in comparison to changes reported for other studies on the effect of GO addition.^7−9^ The presence of GO at GBs provides interfaces for scattering phonons, and additionally, it can inhibit grain growth which results in an increased number of boundaries for the phonon scattering,^27^ and hence a reduction in thermal conductivity can be achieved. However, the consumption of REGO during the sintering process inhibits these positive effects. Therefore, the high thermal conductivity for the samples in the present work could be explained by the relatively high κ_lattice_ of the samples in comparison to the earlier work with GO addition and to the noticeably higher electronic contribution to total thermal conductivity (Figure S6, supporting material), as well as the fact that most of the GO added was actually consumed during sintering.

**Figure 3:**
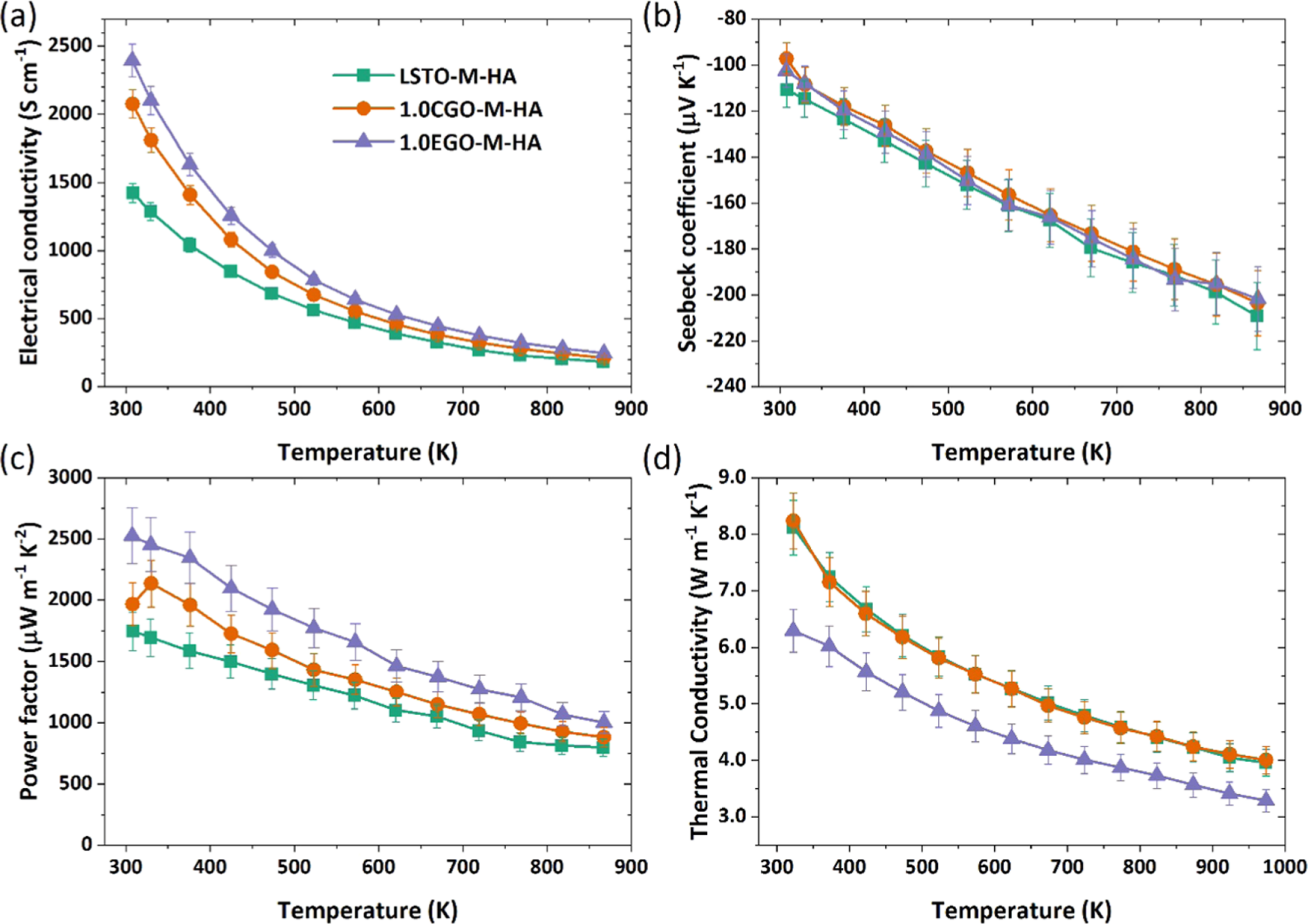
Thermoelectric properties of LSTO samples with conductive grain boundaries; (a) electrical conductivity, (b) Seebeck coefficient, (c) power factor, and (d) thermal conductivity. The samples were sintered under H_2_–Ar flow and in the presence of sacrificial powder.

We now address the effect of graphene addition on LSTO with CGBs, which had been prepared from a hybrid powder of 1.0 wt % EGO or 1.0 wt % CGO and LSTO, where the samples were in contact with the sacrificial powder for sintering. From Raman mapping of 1.0 wt % EGO and LSTO the G band intensity for these samples (example shown in Figure S7, supporting material), the added graphene was entirely consumed after densification. This is consistent with the results above for LSTO prepared with the addition of REGO, which exhibited RGBs (Figure 1), but with complete consumption of the graphene due to the increased sintering time (from 12 to 24 h); the same applied to LSTO with 1.0 wt % CGO samples. The use of a sacrificial powder bed leads to a higher degree of reduction, apparent from the peak shift to lower angles (see Figure S5b, supporting information). It is also clear that there is limited to no peak shift when REGO is used and the samples are prepared without a sacrificial powder bed; the same is observed for EGO addition when the preparation is performed with a sacrificial powder bed. However, for both cases, a slight peak shift to higher angles is observed when GO with a higher level of oxygen content is used. This indicates that use of GO sources plays an important role in the degree of reduction. Therefore, it can be said that using a sacrificial powder bed is essential for preparing samples with a higher level of oxygen vacancies, while it is also essential to choose a GO that has lower oxygen content to maximize the level of reduction. The thermoelectric properties of samples with CGBs are presented in Figure 3. Samples prepared with the addition of 1.0 wt % EGO or 1% CGO exhibit enhanced σ compared to the base LSTO sample (Figure 3a), with EGO performance clearly higher than CGO samples. Electrical conductivity at room temperature increased from 1423 to 2395 S cm^–1^ with EGO addition, an almost 80% increase in conductivity, while it was 2075 S cm^–1^ with CGO addition, which is a nearly 60% improvement. However, improvement in the σ at high temperatures was limited, at 867 K, increasing from 182 to 246 S cm^–1^ and 212 S cm^–1^ with EGO and CGO addition, respectively. Despite the variations in σ, the samples densified in contact with the sacrificial carbon powder show single-crystal-like conduction behavior. These results suggest that the optimized addition of graphene to LSTO ceramics with CGBs could further increase σ. Indeed, Okhay et al.^9^ achieved maximum electrical conductivity at a higher degree of donor doping and a lower level of RGO addition than the values employed in this study, suggesting opportunities for fine tuning to optimize. The reason that EGO addition yields better electrical conductivity than CGO addition is its lower oxygen content (<20 at. %) than that of CGO (∼30 at. %).^37^ Herein, when EGO and CGO were added into the LSTO matrix at the same loading (1.0 wt %), the amount of carbon available to react with the lattice oxygen is higher in EGO than that in CGO, leading to the formation of more oxygen vacancies and thus higher carrier concentration and conductivity.

Similar to the behavior of samples with RGBs, the addition of EGO and CGO to samples with CGBs shows a limited effect on the Seebeck coefficients (Figure 3b). In the temperature range from 300 to 867 K, the *S* values vary from −110 to −209 μV K^–1^ for the LSTO sample, while these are between −103 and −201 μV K^–1^ for the LSTO + EGO (composite) sample and marginally higher for CGO at lower temperatures but within uncertainty limits. The power factor values for the samples with CGBs (Figure 3c) first reflect the single-crystal-like behavior, resulting from processing in sacrificial powder, and second the benefit of adding 1.0 wt % EGO, which improved the σ*S*^2^ values from 1745 to 2525 μW m^–1^ K^–2^ (∼45% improvement in σ*S*^2^); results for CGO addition are intermediate between those for EGO addition and LSTO alone. There is clearly scope for further improvement in the power factor of SrTiO_3_-based ceramics with EGO addition; however, routes to mitigate the densification problems will be necessary for the higher loading levels. Additional milling is certainly necessary, although the effects of the milling procedure on the dispersion and oxidation of graphene need to be further understood.

The thermal conductivity of the samples with CGBs also follows a typical inverse temperature dependency (see Figure 3d); values for the base composition (LSTO) and those with CGO addition are much higher than those for samples with RGBs (Figure 2d), which is mainly due to the increased electronic contribution to κ (see Figure S6, supporting material). The samples prepared with 1.0 wt % EGO shows noticeably lower κ values (Figure 3d), which resulted from the lower lattice contribution. In addition to the (unwanted) increased electronic contribution, the reduction in thermal conductivity with EGO addition and processing with sacrificial powder leads to improved phonon scattering, which was evident in the earlier work on STO-GO composites.^7,9^

We have demonstrated above and in earlier work^34^ that (i) the preparation of LSTO ceramics and composites in contact with a sacrificial carbon powder leads to single-crystal-like electronic conduction behavior in the measured temperature range; (ii) the addition of graphene (REGO/EGO) to the LSTO ceramic matrix (for both RGBs and CGBs) improves the electrical conductivity but without the presence of a powder bed cannot switch the conduction behavior from polycrystalline to single crystal-like; and (iii) the graphene added to the LSTO ceramic is consumed thoroughly via a sacrificial reaction with lattice oxygen to promote the formation of oxygen vacancies. Hence, it is reasonable to propose that the change of polycrystalline to single-crystal-like conduction behavior by muffling is due to its dramatic effect in facilitating the release of oxygen formed through eq 1. Particularly, the requirement for direct contact between the LSTO ceramic and the sacrificial carbon powder suggests that the reaction possibly occurs at the solid/solid interface.^34^ The significant reduction of the LSTO promotes the formation of oxygen vacancies and this reduces the height of the double Schottky barriers at the grain boundaries by the elimination of strontium vacancies at the grain boundaries,^33^ leading to single-crystal-like conduction behavior over the measured temperature range.

**Figure 4:**
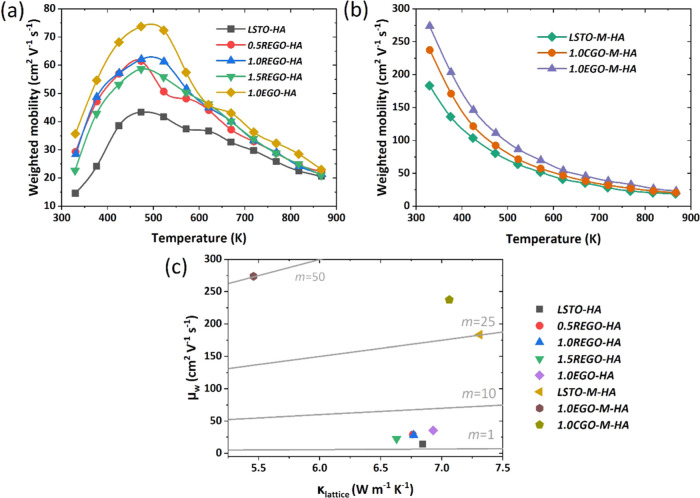
Weighted mobility as a function of temperature for (a) samples with RGBs and (b) samples with CGBs. (c) *m* = μ_w_/κ_lattice_ ratio for both sets of samples with RGBS and CGBs.

To provide greater insight into the transport properties, initially, the mobility, μ, values at room temperature were calculated using the modified Heikes formula (see eq S.1, supporting material and the corresponding text for details), and the results are presented in Figure S.9 in Supplementary material. A mobility of 9.2 cm^2^ V^–1^ s^–1^ at 300 K was reported for La-doped single crystalline SrTiO_3_,^41^ whereas the typical μ value for polycrystalline SrTiO_3_ reaches 1.5 cm^2^ V^–1^ s^–1^ at 300 K.^42^ On the other hand, it was demonstrated that the μ of SrTiO_3_-GO composites could reach to 25.4 cm^2^ V^–1^ s^–1^ at room temperature^33^ due to the faster electron transport without scattering at the GBs. The samples that exhibit RGBs have much lower μ values than the samples with CGBs. Even though the calculated μ values are much lower than those of some other SrTiO_3_-GO composites,^33^ it is clear that the use of a sacrificial powder bed and hence improved reducing conditions is beneficial for the enhancement of the mobility and thus the electrical conductivity. To further confirm the effects of processing conditions on the electronic transport of the samples, the weighted mobility, μ_*w*_, with respect to temperature (*T*) has been calculated for all of the samples using the method of Snyder et al.,^43^eq 4, and experimental electrical resistivity (ρ) and Seebeck coefficient (*S*) values. The results are presented in Figure 4a,b for the samples with RGBs and CGBs, respectively. Samples with RGBs (Figure 4a) exhibit relatively low μ_w_ near room temperature, and the data follow a similar trend to σ with respect to temperature, increasing initially with temperature, reaching a peak value at 473 K, and then decreasing with a further increase of temperature. The highest μ_w_ was 73.8 cm^2^ V^–1^ s^–1^ at 473 K and achieved for the sample with 1.0 wt % EGO addition. Since the μ_w_ is indicative of the quality of the electronic properties, a higher μ_w_ means better electronic performance. Thus, 1.0 wt % is the optimum additive amount for maximizing the electronic performance. In contrast to the data shown in Figure 4a, the samples with CGBs had significantly higher μ_w_ values than the samples with RGBs; simply by changing the processing, the μ_w_ of the base LSTO material was enhanced from 14.6 to 183.5 cm^2^ V^–1^ s^–1^ at 330 K. The effect of graphene addition was even more pronounced for the samples with CGBs, reaching a μ_w_ value of 273.7 cm^2^ V^–1^ s^–1^ at 330 K for the sample prepared with 1.0 wt % EGO addition.
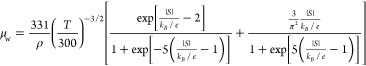
4

The potential for high thermoelectric performance materials can also be assessed through the thermoelectric quality factor *B*, which is proportional to the ratio of the weighted mobility to lattice thermal conductivity (μ_w_/κ_lattice_).^44^ Therefore, a high *B* or μ_w_/κ_lattice_ ratio is desirable for high-performance thermoelectric materials since their electronic properties and thermal properties are characterized by μ_w_, and κ_lattice_, respectively. Based on our results (Figure 4c), the samples with CGBs exhibit much superior performance to the samples with RGBs. The μ_w_/κ_lattice_ ratio was quite low for the samples with RGBs (mainly less than 10) and increased tenfold for the samples with CGBs. Since the materials in this investigation exhibited high κ_lattice_ values, as high as 7.3 W m^–1^ K^–1^, it is clear that a further reduction in κ_lattice_ is a priority to enhance *zT* values.

**Figure 5:**
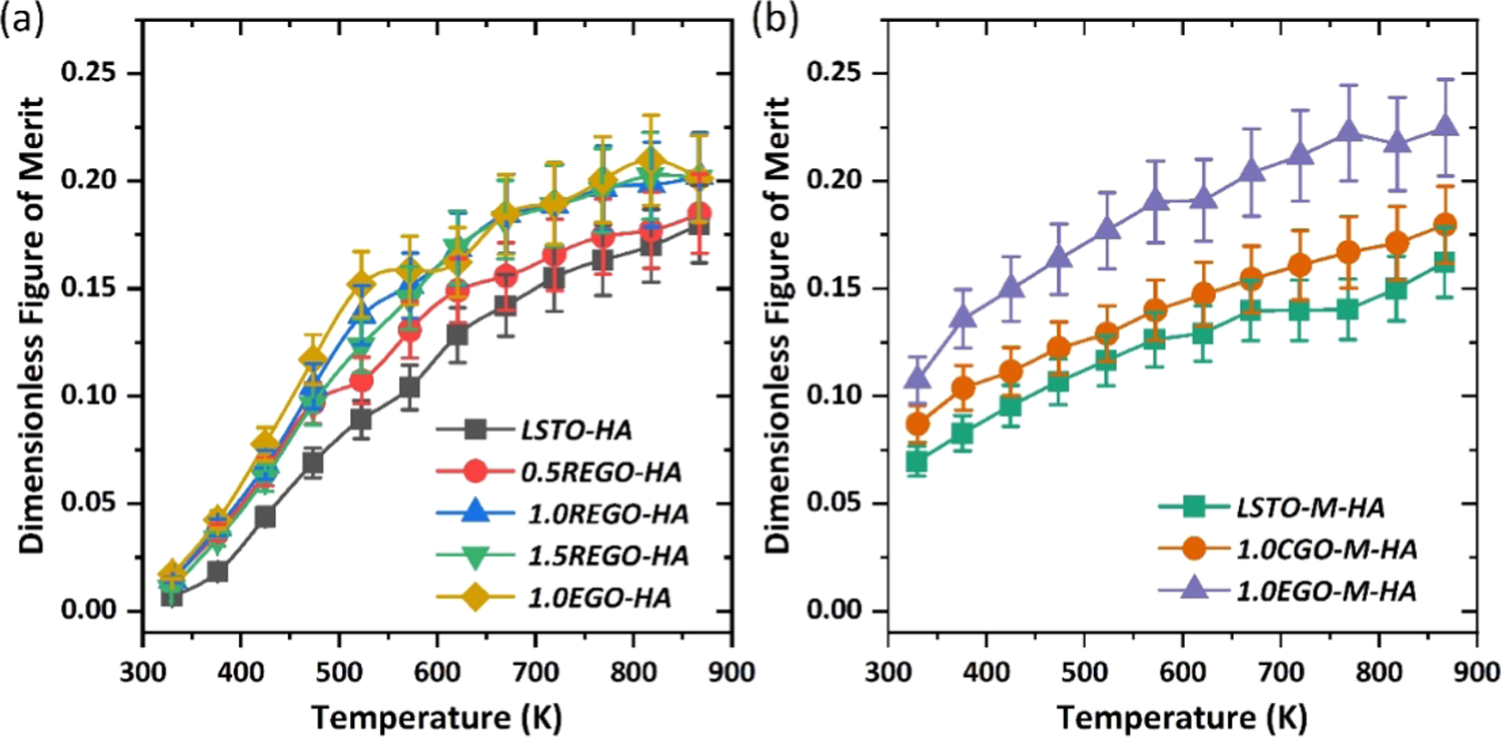
Dimensionless figure of merit for LSTO-based composites: Samples with (a) RGBs and (b) CGBs.

The overall performance of the ceramics with both RGBs and CGBs was evaluated in terms of the dimensionless figure of merit, *zT*. Figure 5a shows the effect of graphene addition on *zT* for samples with RGBs. The addition of graphene to LSTO with RGBs increased the *zT* values, although there was very limited improvement in *zT* near room temperature; the improvement in *zT* was the greatest at high temperatures. The maximum *zT* only increased from 0.18 to 0.20 at 867 K with addition of ≥1.0 wt % EGO/REGO. As the addition of graphene to LSTO with RGBs cannot change the conduction behavior from grain boundary controlled to single crystal-like, there is no widening of the thermal window in this set of samples. On the other hand, the addition of graphene to the dense samples with CGBs enhances *zT* values (Figure 5b); compared to LSTO, 1.0 wt % EGO addition increases *zT* from 0.07 to 0.11 at 330 K (54% enhancement) and from 0.16 to 0.22 at 867 K (39% enhancement). Once again, the results for CGO samples are intermediate between *zT* values for LSTO and EGO but much closer to those for LSTO. Another clear feature of Figure 5b is that the average *zT*, over the range of almost 540 K, also increased by 50%, indicating that the addition of graphene, combined with a processing route to give CGBs, significantly improves the thermoelectric properties of LSTO ceramics. Although the power factor values achieved in this work are comparable with those reported in our earlier investigation,^7^ the overall *zT* values in the present work are much lower because of the high thermal conductivity (∼7 W m^–1^ k^–1^ at room temperature). However, the *zT* values achieved in this work are comparable to other works on donor-doped STO-GO composites with similar doping concentrations, where maximum *zT* was below 0.20 within the same temperature range.^9^ Nanostructuring of the LSTO ceramics (e.g., grain size reduction) and/or employing a complex doping strategy could potentially lead to further enhancement of *zT* values.

**Figure 6:**
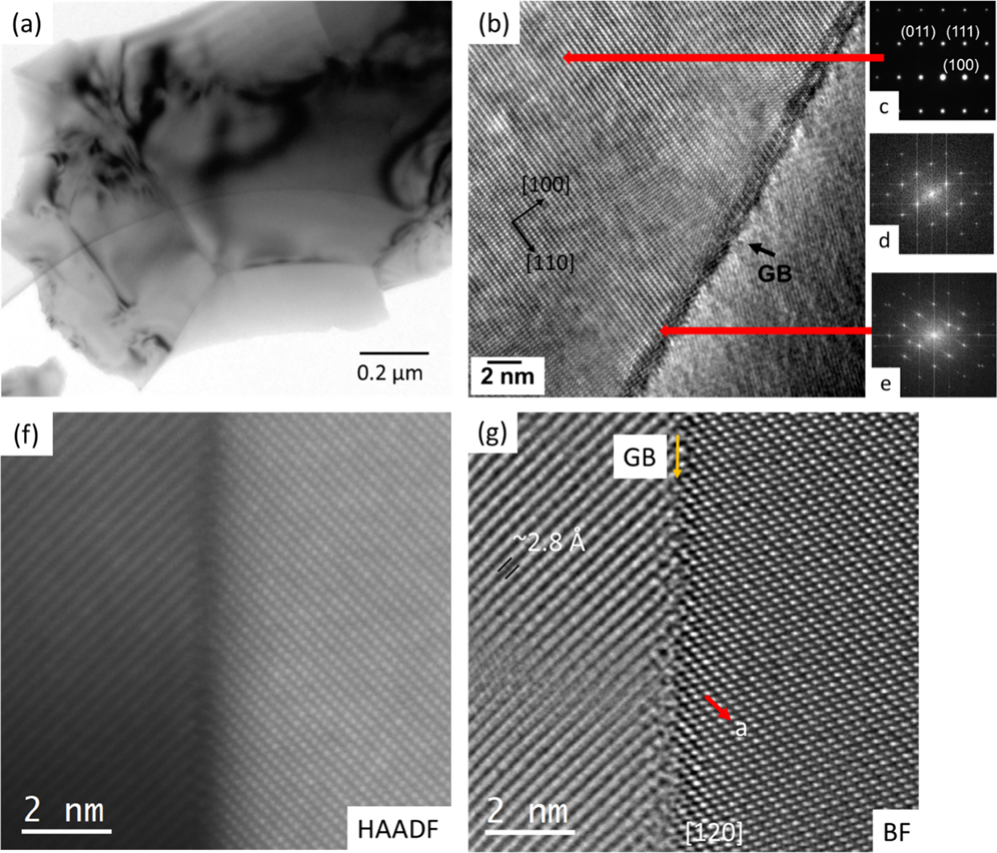
(a) TEM image of high power factor 1.0EGO-M-HA sample showing grain boundaries. (b) HRTEM image for a grain boundary presented in (a). (c, d) Related SAED and FFT data from the upper grain areas in (b) and (e) FFT data from the grain boundary in (b). (f) HAADF and (g) BF images of the grain boundary.

TEM and selected area electron diffraction (SAED) were employed to further understand the high power factor samples 1.0EGO-M-HA. Low-magnification TEM images showed clean grain boundaries (e.g., Figure 6a). SAED patterns for the samples were collected along [001]p (“p” denotes perovskite), [110]p, and [111]p zone axes to provide details of the crystal structure of the sample (Figure S8, supporting material). The diffraction patterns confirm the cubic structure; this is consistent with the XRD observations (Figure S3). Subsequently, a more detailed electron microscopy study focused on the characterization of the grain boundaries. An HRTEM image for the 1.0EGO-M-HA sample with a grain boundary is shown in Figure 6b–e. The grain on the left-hand side of the image is oriented along the [110] zone axis (Figure 6c). The related SAED and FFT data from the grain area do not show any extra or diffuse reflections (Figure 6d,e). However, the FFT data from the grain boundary shows streaking along with the main reflections. This could be an indication of a change of lattice parameters (i.e., change in the composition at the grain boundary). To confirm this hypothesis, atomic resolution STEM-HAADF-BF-EELS data were collected from the grain boundaries; STEM-HAADF and BF images for a grain boundary are presented in Figure 6f,g. The grain on the left-hand side of the image has been viewed along the [120] zone axis. A spacing of ∼2.8 Å for the lattice fringes observed for the grain on the left-hand side suggests that the grain orientation is close to the major zone axis. It can be seen that the grain boundary is clean without any evidence of a grain boundary phase.

**Figure 7:**
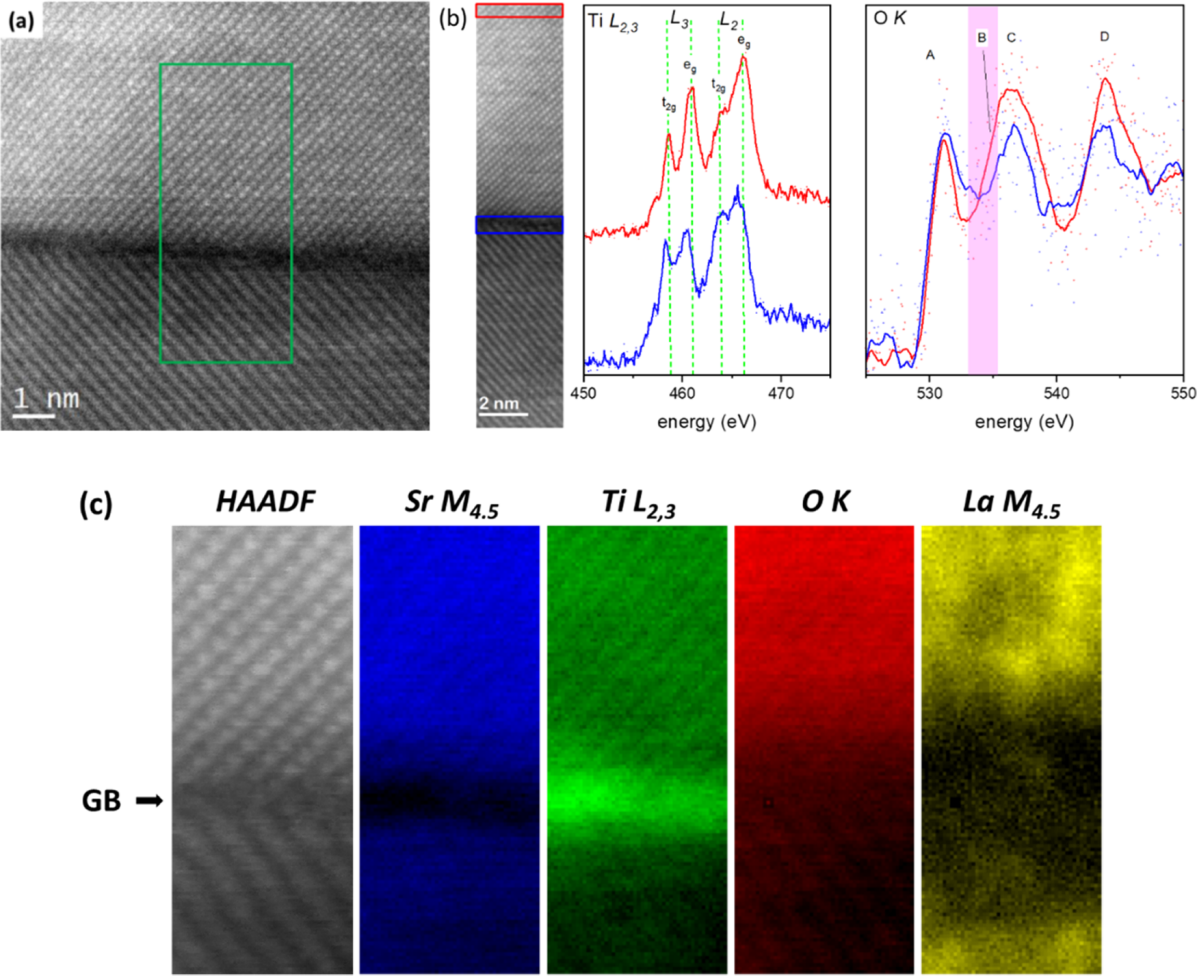
(a) HAADF image of the grain boundary. EELS data were collected from the area denoted by the green rectangle. (b) Ti L_3,2_ and O K spectra obtained from the grain (integration over the rectangular red box) and grain boundary (blue box) in the smaller HAADF image. The spectra are background-subtracted but otherwise not normalized or scaled to allow for a direct comparison. Raw data are shown as a scatter plot, with overlaid solid lines displaying smoothed spectra (Savitzky–Golay, 20 data points) as a guide to the eye. (c) HAADF signal during data acquisition and EELS maps for Sr, Ti, O, and La from the (green box) region shown in (a).

Figure 7 shows the high-resolution electron microscopy data for a single grain boundary in the 1.0EGO-M-HA sample. STEM-EELS data from the grains and their boundary were collected from the region denoted by the green rectangle in Figure 7a; the HAADF signals, acquired simultaneously with EELS data (Figure 7b), are shown in Figure 7c. The resulting EELS maps show distinct variations of the elemental distribution at grains and grain boundaries (Figure 7c). There is a pronounced depletion of Sr at the grain boundary with a depth of depletion of about 1 nm (corresponding to ∼2 atomic planes on each side of the interface), while relative depletions of O and La are also evident. The EELS spectra for Ti L_3,2_ and O K for the (bulk) grain and the grain boundary regions are presented in Figure 7b. The fine-structure Ti L_3,2_ EELS spectra consist of four peaks, namely, the L_3_ t_2g_, e_g_, and L_2_ t_2g_, e_g_ peaks arising from 2p_3/2_ →3d and 2p_1/2_ → 3d transitions, respectively. The energy positions, relative splitting, and intensity ratios of the peaks are sensitive to the local Ti–O coordination and bonding environment.^45^ As can be seen in Figure 7b, there is a relative shift of the L_3_ t_2g,_ e_g_ and L_2_ t_2g_, e_g_ toward lower energy losses at the boundary (vertical green lines act as a guide to the eye), as well as a change in the relative t_2g_–e_g_ splitting and intensity ratios across the grain boundary region.^46−48^ These characteristic changes in the Ti *L*_3,2_ fine structure are indicative of localized changes in the bonding environment of Ti at the interface, namely, of the presence of Ti^3+^ and oxygen vacancies in the grain boundary region.^45,49^ This observation is corroborated by changes of the fine structure of the O K edge at the interface. The O K edge shows two main peaks (Figure 7b) marked as A and B–C (the B–C spitting is not clearly resolved in this experiment due to the choice of energy dispersion to capture all edges simultaneously so that the position of the feature commonly denoted peak B in the analysis of the fine structure of O K edges of SrTiO_3_ appears here only as a shoulder on the low energy loss side of peak C and is marked by a magenta band in Figure 7b). Peaks A and B correspond to unoccupied states arising from O-2p to Ti-3d orbital hybridization (t_2g–e_g__ splitting), while peaks C and D correspond to the hybridization of O-2p orbitals with Sr-4d empty states. An additional peak labeled D is commonly linked to hybridization with Sr-4s and 4p states; however, due to its distance from the edge onset, a precise interpretation can be challenging. As can be seen in Figure 7b, the broadening of peak A and the relative intensity drop of B at the interface (the spectra are not normalized and displayed on the same intensity scale for a meaningful intensity comparison) indicate a relative reduction of Ti from Ti^4+^ to Ti^3+^, in agreement with the Ti L_2,3_ data. Furthermore, the observed drop of the intensity of the C and D peaks at the grain boundary is in agreement with the observed lower concentration of Sr at the interface. In summary, atomic resolution imaging and chemical mapping provide direct evidence for the presence Ti^3+^ and oxygen vacancies at the interface, which will contribute as additional charge carriers in the material and explain the origin of the higher electrical conductivity at the grain boundaries.

**Figure 8:**
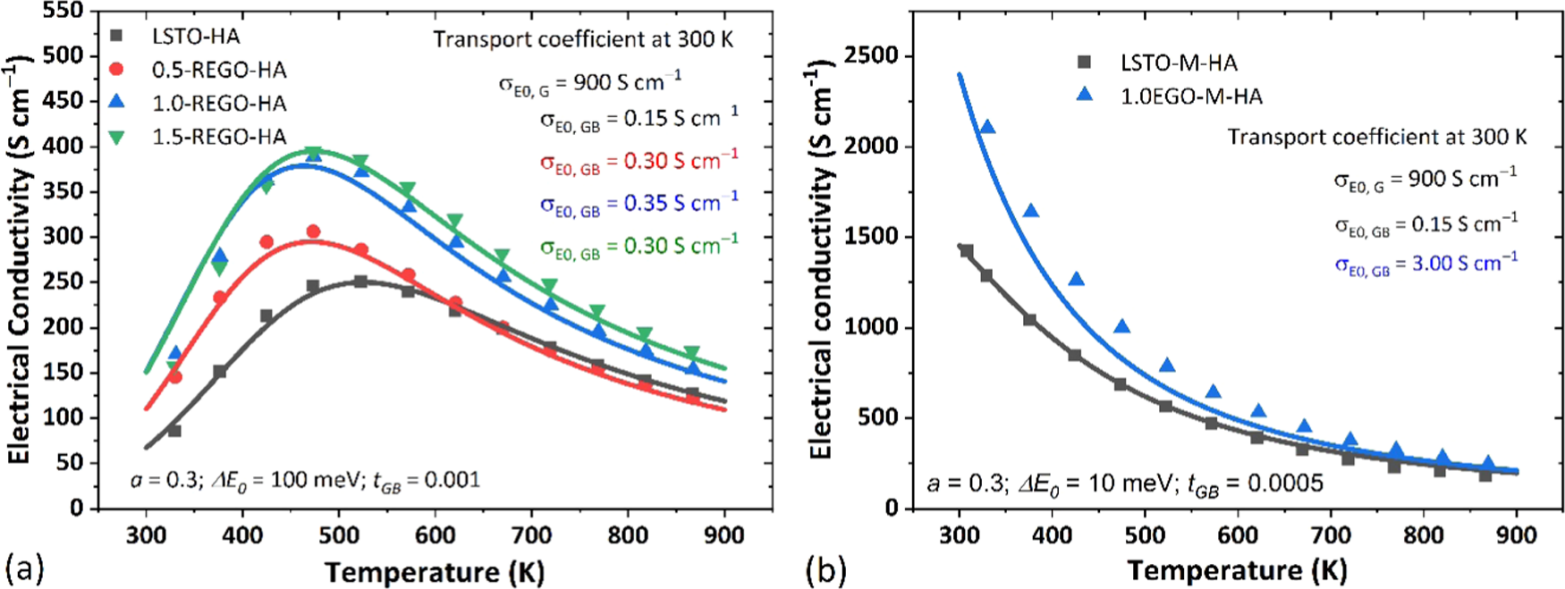
Experimental electrical conductivity data (solid symbols) and results from two-phase model fitting (solid lines) for (a) LSTO-REGO composite samples prepared without a carbon sacrificial bed and (b) LSTO-EGO samples prepared with a sacrificial carbon bed. The fitting parameters employed are presented in Table S1 (supporting material).

We recently demonstrated^34^ that the change of the electronic conduction behavior of SrTiO_3_-based ceramics from thermally activated to single crystal-like can be accurately described by a two-phase model where the barrier height at the GBs is used as a fitting parameter. In the present work, we further modeled the effects of graphene addition on the conduction behavior of LSTO via the two-phase model.^32,50^ The modeling and computational procedures are detailed in our earlier work;^34^ the results are presented in Figure 8. We omitted CGO samples from this analysis as their results are intermediate between those for LSTO and EGO with CGBs (Figures 3, 4b, and 5b) and provide no further insight. Two sets of modeling parameters were used to simulate the temperature dependencies of conductivity for the two groups of samples that exhibited thermally activated and single-crystal-like conduction behavior, respectively. The modeling parameters employed are presented in Table S1 and Figure 8. For each set of modeling parameters, the transport coefficient of the grain boundary phase (σ_*E*_0_,GB_) at 300 K was the only fitting parameter used to simulate the effect of graphene addition on the electrical conductivity. In brief, at a given temperature, the transport coefficient is proportional to weighted mobility;^43^ therefore, a higher σ_*E*_0_,GB_ indicates enhanced carrier mobility in the grain boundary phase.

For the samples containing different levels of REGO and prepared without a sacrificial carbon bed (thermally activated electrical conductivity, Figure 8a), σ_*E*_0__ at 300 K increases from 0.15 to 0.35 S cm^–1^ with 1.0 wt % REGO addition but σ_*E*_0_,GB_ reduces slightly to 0.30 S cm^–1^ when REGO addition reaches 1.5 wt %. This indicates a clear improvement in the conduction behavior of GBs with REGO addition and can be explained by the consumption of REGO during high-temperature sintering (confirmed by Raman results in Figure 1), promoting the reduction of grain boundaries. In contrast, for composites prepared with a sacrificial carbon bed, σ_*E*_0_,GB_ increased from 0.15 to 3.0 S cm^–1^ with only 1.0 wt % EGO addition. This is a 20-fold improvement in σ_*E*_0_,GB_ and presents a significantly increased σ_*E*_0_,GB_ in comparison to samples prepared without a sacrificial carbon bed, indicating much higher carrier mobility at GBs for these samples.

The HAADF TEM evidence in Figure 7 showed that there was a much higher concentration of Ti^3+^ and oxygen vacancies at the GBs than that in bulk grains when a sacrificial carbon bed was used. In combination with STEM-EELS results, this showed that the use of a sacrificial bed in addition to GO addition is an effective approach to prepare samples with highly reduced GBs that exhibit higher electrical conductivity and single-crystal-like temperature-dependent conductivity.

## 4 Conclusions

The effects of processing conditions and the addition of graphene on the thermoelectric performance of La-doped SrTiO_3_ have been systematically investigated, allowing the role of the two approaches to be separated. It has been demonstrated that the addition of graphene, regardless of the preparation method (i.e., chemical or electrochemical), is beneficial for improving the performance of LSTO ceramics. There is clear evidence that EGO yields higher performance than CGO addition. However, CGO also improves the overall performance of LSTO ceramics, but the properties obtained are in between those of LSTO ceramics and LSTO + EGO composites. Furthermore, the simple addition of REGO without muffling cannot eliminate the grain boundary-controlled electron conduction and thermally activated behavior is observed below 450 K. However, it was apparent that this improvement was enhanced through the production of conductive grain boundaries. Structural characterization of GBs and modeling work based on two-phase material showed that the increase in the oxygen vacancy concentration at the GBs and the resulting improvement in the carrier mobility via observed changes in σ_*E*_0__ promotes superior electronic transport properties. As a result, a high power factor of 2525 μW m^–1^ K^–2^ was achieved at 300 K with 1.0 wt % EGO addition when the ceramics were prepared with muffling and under H_2_–Ar flow. The weighted mobility values showed that processing with sacrificial powder and addition of graphene significantly enhances the electronic performance of the LSTO ceramics, confirming the structural characterization and modeling results.

Since the added graphene is almost entirely consumed via the sacrificial reaction with lattice oxygen to create oxygen vacancies, its role in reducing thermal conductivity via interface phonon scattering was limited. The μ_w_/κ_lattice_ ratio was improved tenfold once the samples had CGBs in comparison to the case for RGBs. Indeed, these higher μ_w_/κ_lattice_ values are comparable with results for a number of metallic thermoelectrics^51^ and suggest that such oxides are worthy of further development to overcome limitations imposed by thermal transport. Although the high thermal conductivity limited the maximum *zT* value of the present materials to 0.22 at 867 K (for 1.0 wt % EGO-added sample), the increase in *zT* and the noticeable improvement in average *zT* indicate that this processing approach and the addition of graphene are beneficial for optimizing the thermoelectric performance of LSTO ceramics. These results suggest that reducing the lattice thermal conductivity, either by nanostructuring or a complex doping strategy, is a priority and could lead to much higher *zT* values for SrTiO_3_-based thermoelectric materials.

## Supporting Information Available

SEM micrographs, XRD patterns, lattice, and electronic contribution to total thermal conductivity; Raman spectra, SAED patterns, modeling parameters, and carrier mobility calculations (PDF)
