# Supplementary material for: Controlling the Thermoelectric Behavior of La-Doped SrTiO3 through Processing and Addition of Graphene Oxide
Source: ACS Appl Mater Interfaces. 2022 Nov 22;14(48):53711–23. doi: 10.1021/acsami.2c14408 (PMC9743083; doi:10.1021/acsami.2c14408)
Supplement: Supplementary file 1 — am2c14408_si_001.pdf [file am2c14408_si_001.pdf]

Supporting Information

Controlling the thermoelectric behaviour of La-doped $SrTiO_3$ through processing and the addition of graphene oxide

Dursun Ekren[1,2,§], Jianyun Cao[1,3,§], Feridoon Azough[1], Demie Kepaptsoglou[4,5], Quentin Ramasse[4,6], Ian A. Kinloch[1,7], Robert Freer[1,*]

[1] Department of Materials, University of Manchester, Oxford Road, M13 9PL, United Kingdom

[2] Department of Metallurgical and Materials Engineering, Iskenderun Technical University, 31200, Hatay, Turkey.

[3] Key Laboratory of LCR Materials and Devices of Yunnan Province, School of Materials Science and Energy, Yunnan University, Kunming 650500, China

[4] SuperSTEM Laboratory, SciTech Daresbury Campus, Daresbury WA4 4AD, United Kingdom

[5] Department of Physics, University of York, York YO10 5DD, United Kingdom

[6] School of Chemical and Process Engineering, University of Leeds, Leeds LS2 9JT, United Kingdom

[7] Henry Royce Institute and National Graphene Institute, University of Manchester, Oxford Road, M13 9PL, United Kingdom

*Corresponding author.

E-mail address: Robert.Freer@manchester.ac.uk

[§] These authors contributed equally to this work.

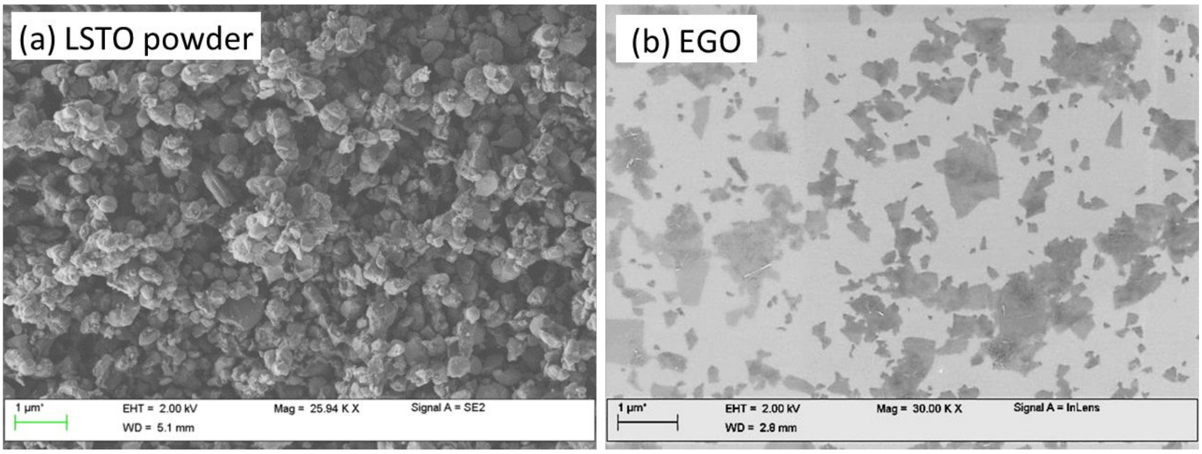

Figure S1 SEM micrographs for (a) LSTO powder after the calcination process. and (b) electrochemically (EGO) exfoliated graphene oxide flakes

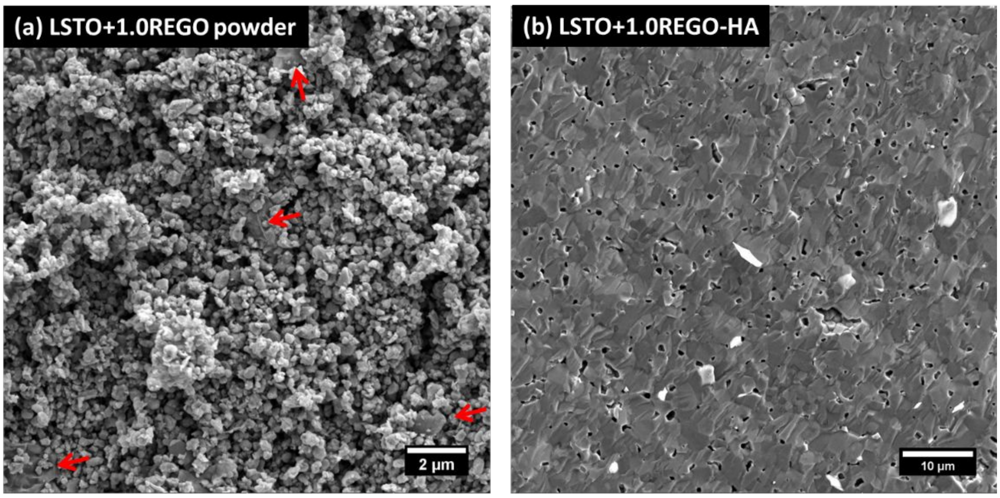

Figure S2 SEM micrographs for LSTO+1.0REGO (a) powder and (b) bulk sample obtained after densification without use of muffling. This figure indicates the consumption of (R)EGO during the densification process

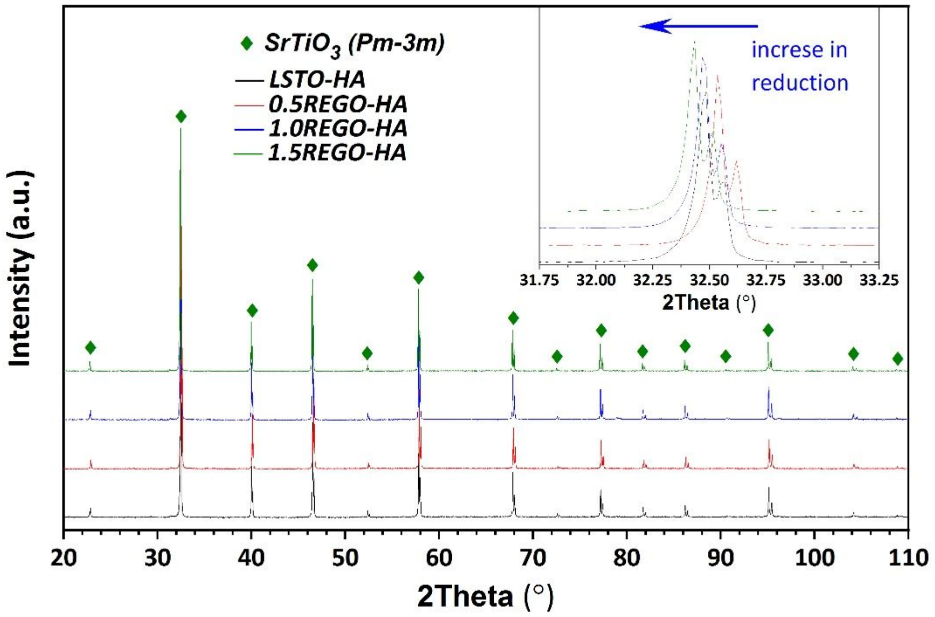

Figure S3 XRD patterns for LSTO samples with resistive grain boundaries, showing the effect of the amount of graphene addition on the crystal structure.

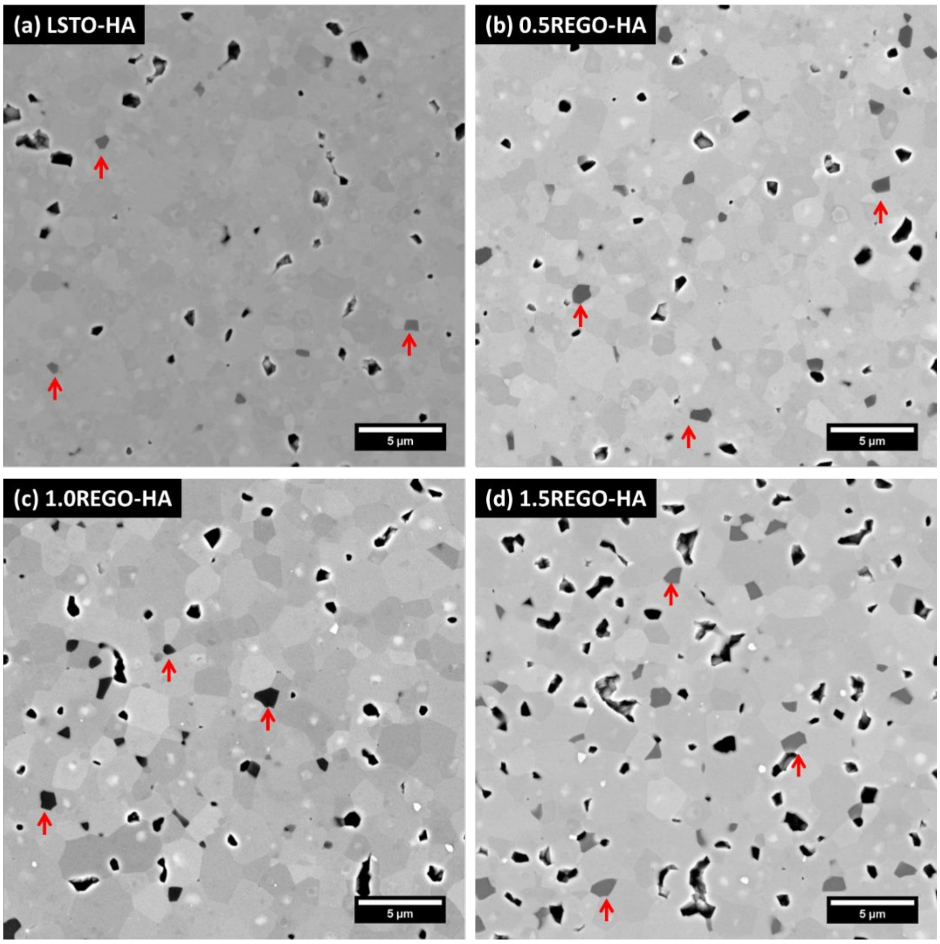

Figure S4 SE-SEM images for LSTO samples prepared with different REGO additions; (a) base composition, (b) 0.5 wt% REGO and (c) 1.0 wt% REGO and (d) 1.5 wt% REGO. Red arrows indicate Ti-rich secondary phases.

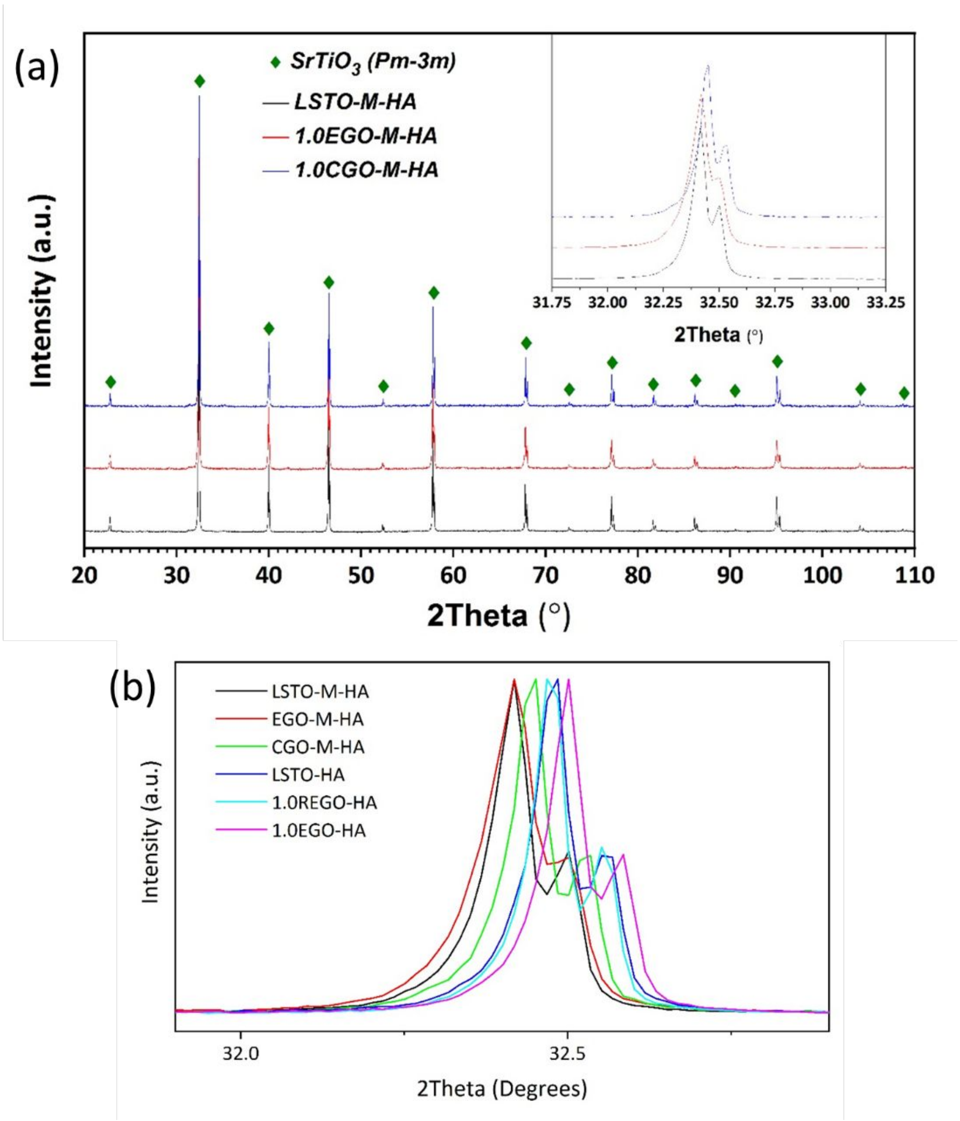

Figure S5 (a) XRD patterns for LSTO samples with conductive grain boundaries, showing the effect of different types of graphene on the crystal structure. (b) XRD pattern for the (110) peak for the samples prepared with/without the use of sacrificial powder bed

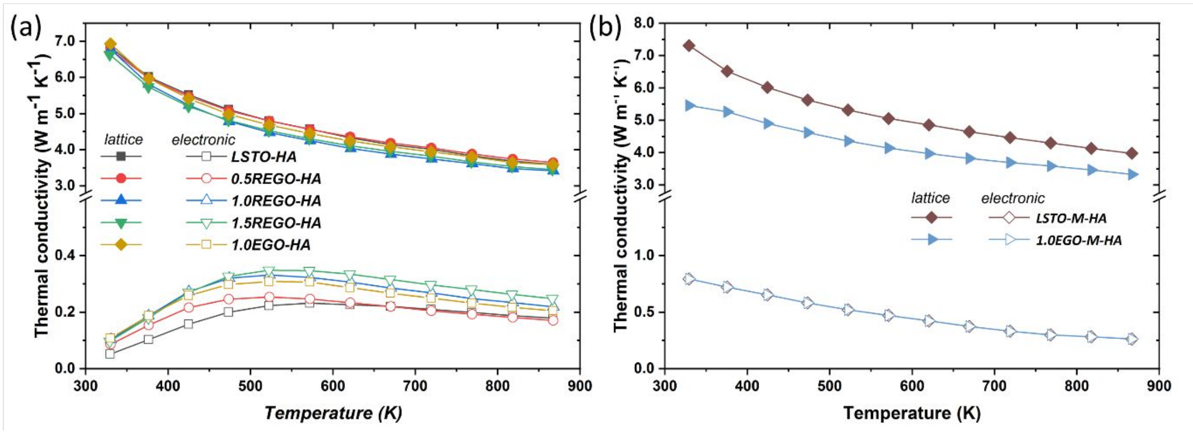

Figure S6 Lattice and electronic contribution to total thermal conductivity of (a) samples with resistive grain boundaries, and (b) samples with conductive grain boundaries.

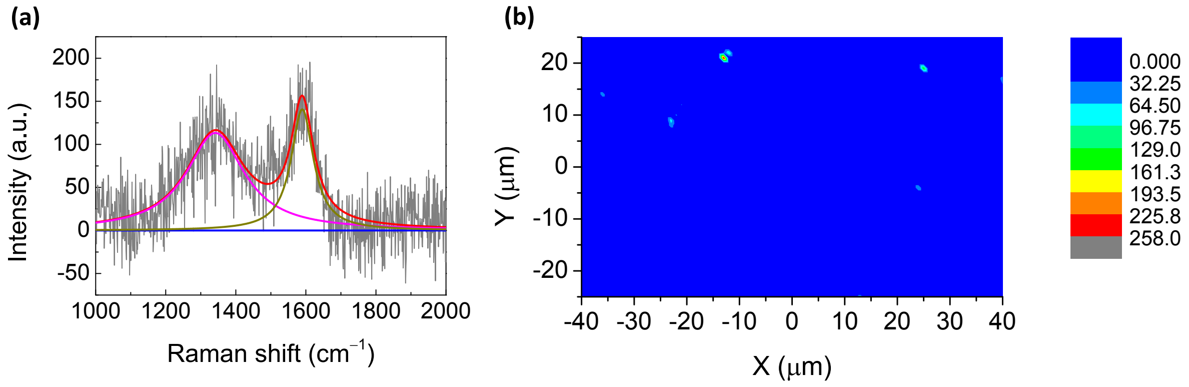

Figure S7 Raman spectroscopy characterization of LSTO ceramic densified from hybrid powder of 1.0 wt.% EGO and LSTO. (a) Typical Raman spectrum showing D and G band peaks, which confirms the presence of EGO after the sintering process. (b) G band intensity mapping of the bulk composite that shows the consumption of EGO during the sintering process.

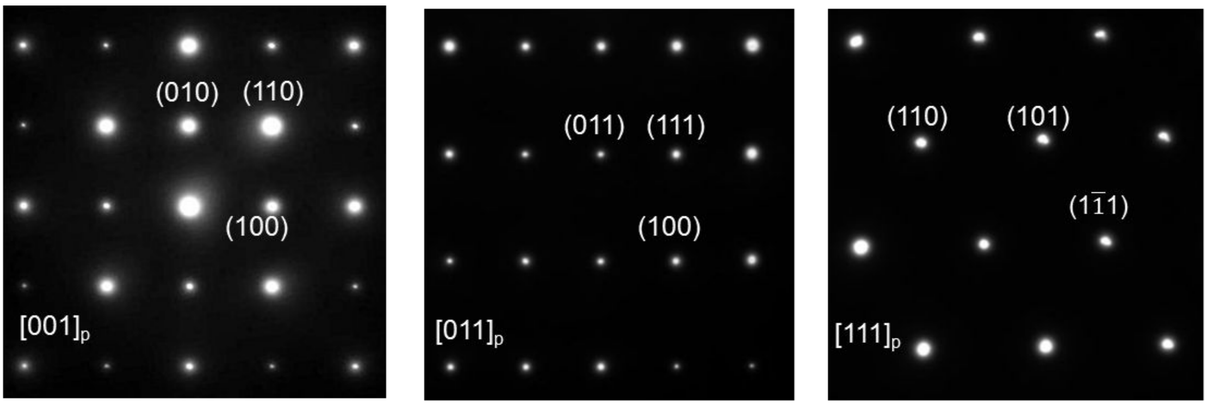

Figure S8 SAED patterns along the major zone axes ($[001]_p$, $[011]_p$ and $[111]_p$) for the high power factor, 0.1EGO-M-HA sample.

Table S1 Modelling parameters for $La_{0.08}Sr_{0.9}TiO_{3-\delta}$ (LSTO) ceramics prepared under different sintering conditions and at different amounts of graphene addition.

<table>
<tr><th rowspan="2">Sample</th><th rowspan="2">t_{GB} *</th><th colspan="2">Band offset function</th><th colspan="2">Transport coefficient (300 K)</th></tr>
<tr><th>a</th><th>ΔE_0</th><th>$\sigma_{E_{0,G}}$</th><th>$\sigma_{E_{0,GB}}$</th></tr>
<tr><td>LSTO-HA</td><td>0.001 (1.75 μm)</td><td rowspan="6">0.3</td><td rowspan="4">100 meV</td><td rowspan="6">900 S cm^{-1}</td><td>0.15 S cm^{-1}</td></tr>
<tr><td>0.5-REGO-HA</td><td>0.001 (3,40 μm)</td><td>0.30 S cm^{-1}</td></tr>
<tr><td>1.0-REGO-HA</td><td>0.001 (4.48 μm)</td><td>0.35 S cm^{-1}</td></tr>
<tr><td>1.5-REGO-HA</td><td>0.001 (3.77 μm)</td><td>0.30 S cm^{-1}</td></tr>
<tr><td>LSTO-M-HA</td><td>0.0005 (2.67 μm)</td><td rowspan="2">10 meV</td><td>0.15 S cm^{-1}</td></tr>
<tr><td>1.0-EGO-M-HA</td><td>0.0005 (3.88 μm)</td><td>3.00 S cm^{-1}</td></tr>
</table>

* The average grain size of the sample is shown in the parenthesis.

It is possible to estimate the carrier concentration values of a perovskite structured material using modified Heikes formula [1] given by Equation S.1 with the help of experimental electrical conductivity and the Seebeck coefficient values. Then, Equation S.2 can be used to calculate the carrier mobility through its relationship with electrical conductivity and carrier concentration. Using this approach, the mobility values of the samples were calculated and presented in Figure S.9.

$$n = \frac{4}{V}\left\{\frac{1}{exp\,(\pm Se/k) + 1}\right\} \tag{S.1}$$

$$\sigma = ne\mu \tag{S.2}$$

where, n is carrier concentration; V is the unit cell volume; S is the Seebeck coefficient; e is elemental charge; k is the Boltzmann constant; σ is electrical conductivity and μ is carrier mobility.

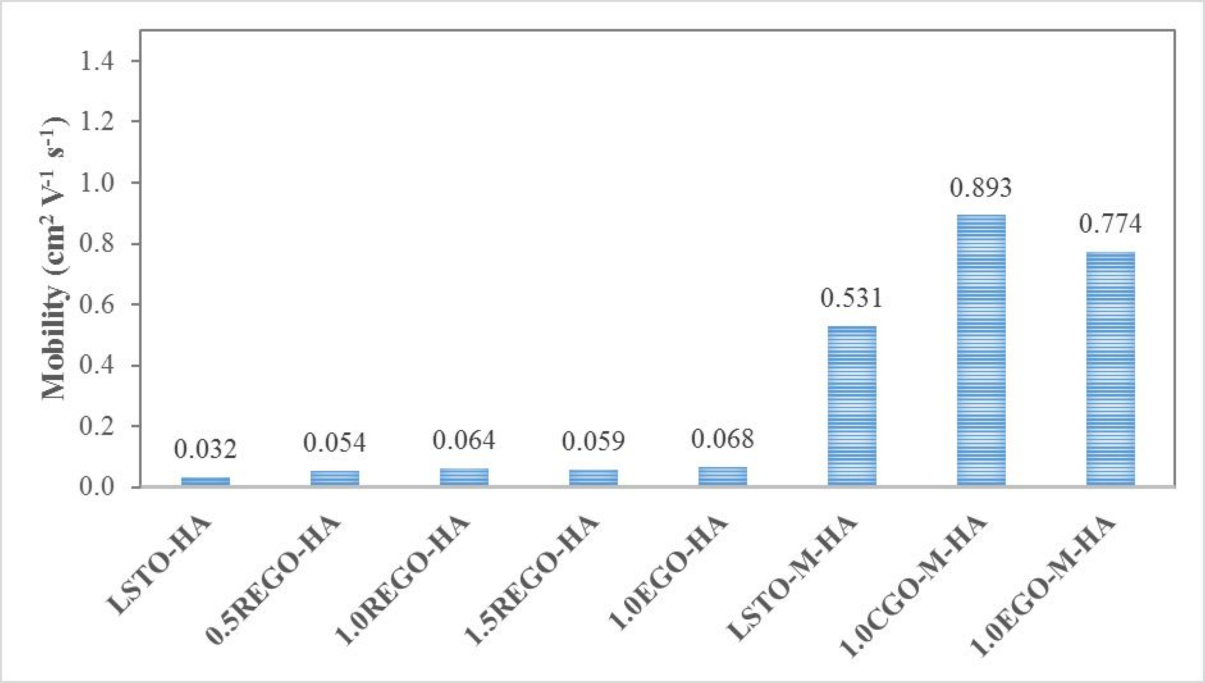

Figure S.9 Mobility values for the samples calculated using modified Heikes formula.

References

(1) Taguchi, H.; Sonoda, M.; Nagao, M. Relationship between Angles for Mn-O-Mn and Electrical Properties of Orthorhombic Perovskite-Type $(Ca_{1-x}Sr_x)MnO_3$. *J. Solid State Chem.* **1998**, *137* (1), 82–86. https://doi.org/10.1006/jssc.1997.7701.
